# The Additional Value of Endolymphatic Hydrops Imaging With Intratympanic Contrast for Diagnostic Work-Up—Experience From a Neurotology Center in Austria

**DOI:** 10.3389/fsurg.2021.672865

**Published:** 2021-10-22

**Authors:** Lennart Weitgasser, Anna O‘Sullivan, Alexander Schlattau, Sebastian Roesch

**Affiliations:** ^1^Department of Otorhinolaryngology, Head and Neck Surgery, Paracelsus Medical University, Salzburg, Austria; ^2^Institute of Experimental Neuroregeneration, Paracelsus Medical University, Salzburg, Austria; ^3^Department of Radiology, Paracelsus Medical University, Salzburg, Austria

**Keywords:** hydrops, imaging, dizziness, vertigo, Menière, magnetic resonance imaging, intratympanic

## Abstract

**Objective:** To illustrate the merit of hydrops imaging during clinical workup of dizziness and balance disorders.

**Background:** Ever since the first description of *in-vivo* endolymphatic hydrops imaging in 2007, this diagnostic tool has been implemented in an increasing number of centers. The more experience in its clinical application is gathered, the more it is possible to critically assess its potential value for the diagnostic workup. This article intends to provide information about the experience of handling and utilization of endolymphatic hydrops imaging in one of the first centers in Austria.

**Methods:** Retrospective analysis and review of clinical cases.

**Results:** Based on our experience of endolymphatic hydrops imaging (EHI), which was established in cooperation between our departments of radiology and otorhinolaryngology in 2017, we have exclusively used intratympanic application of a contrast agent prior to magnetic resonance imaging, as this approach provides high quality imaging results. In 42.6% of cases, EHI could lead to the diagnosis of MD or HED. Since precise vestibular examination is still necessary, EHI is not a tool to replace the clinical examination but rather to add significantly to the interpretation of the results.

**Conclusion:** Endolymphatic hydrops imaging represents a valuable, safe and well-applicable tool for evaluating cases with inconclusive clinical results. However, its potential additional diagnostic benefits rely on a correct indication based on prior thorough vestibular investigations.

## Introduction

The diagnostic work-up of recurrent vestibular disorders still represents a challenging task, due to their variable clinical presentation or inconsistent diacritic results. Symptomatic and historically based classification systems may be helpful tools, especially in Menière's disease (MD) with its classical triad of rotatory vertigo, low-frequency hearing loss as well as tinnitus or aural fullness. However, in several cases symptoms do not present themselves in a way such that a satisfactory conclusion is possible. Vestibular and cochlear manifestations may occur completely independent from each other but also monosymptomatic forms of the disease seem to be existent.

Endolymphatic hydrops represents the anatomical correlate of a multifaceted clinical picture and provides a common ground for different perspectives on the actual cause and pathophysiology in the context of MD and diseases of the human labyrinth.

Ever since the first description of the visualization of endolymphatic hydrops in 2005 by Zou et al. (1), the scientific interest and the number of publications in this field have been constantly growing.

Contrast-enhanced, high-resolution magnetic resonance imaging (MRI) is capable of visualizing the endolymphatic hydrops (ELH) *in-vivo*, which reveals the pathophysiological basis of the clinical syndrome originally described by Prosper Menière (2–4). Based not only on symptomatic but also on imaging characteristics, a new terminology was proposed (5, 6) to sum up this spectrum of disorders as “Hydropic Ear Disease” (HED).

In our clinic, we began performing specific MRI (named “hydrops MRI”) in patients with suspected ELH in 2017. Before this time, we generally referred to typical symptoms of MD and occasionally used electrocochleography for the diagnostic work-up. The introduction of hydrops MRI has enabled us to improve the diagnostic accuracy and subsequent care for patients with recurrent vestibular symptoms.

The purpose of this article is to illustrate the establishment and application of hydrops imaging at a tertiary neurotology referral center and critically evaluate its potential benefits for diagnostics.

## Materials and Methods

### Course of Action and Decision-Making Prior to Hydrops Imaging

The selection of patients for hydrops imaging at our center is based on the following criteria and preconditions:

Potential candidates are adult patients, presenting with typical symptoms, suspicious for HED, according to the 1995 AAO-HNS Guidelines and the Equilibrium Committee Amendment (7, 8). Clinical presentation is supposed to include recurrent episodes of rotatory vertigo, lasting for minutes to hours and/or aural symptoms like fullness, tinnitus and sensorineural hearing loss (SNHL), ideally audiometrically documented. However, as it is well-known that symptoms may occur completely independent from each other and to a variable in extend, a simultaneous presence cannot be expected in every patient. Therefore, the diagnostic evaluation may not be straight-forward.

Patients chosen for hydrops imaging are those with symptoms, suspicious of ELH, indecisive diagnostic results and first referral at our center. However, patients with distinct clinical symptoms for years, clearly fulfilling diagnostic criteria for MD, and comprehensive diagnostic results do not qualify for hydrops imaging, since additional diagnostic value for the individual is not expected.

The first diagnostic step for this group of patients remains history taking and distinct vestibular examination. Other causes of vestibular dysfunction or vestibular disease, as well as diseases of the central nervous system initially need to be excluded. We therefore rely on the execution of the HINTS exam, followed by subsequent otoscopy, audiometric and vestibular investigations, such as videonystagmography (VNG), video-head-impulse-test (vHIT) and vestibular evoked myogenic potentials (VEMPs) testing. Moreover, neurological and, if necessary, psychological investigations are performed by the appropriate departments.

### Pre-interventional Arrangements

At our center, the application of the contrast medium for displaying the perilymphatic space during the MRI is administered exclusively intratympanically. Therefore, the definition of an “index ear” is mandatory. This definition is based on previous clinical and diagnostic findings, which consist of laterality of low-frequency hearing loss, tinnitus or aural fullness, missing caloric response during VNG and/or decreased VEMPs. In case of bilateral symptoms, the side more severely affected is selected.

Clinical circumstances representing a contraindication for intratympanic administration of any agent, such as acute otitis media or recent trauma or surgery, need to be excluded prior to application.

Informed consent is obtained, including specifically, the information about an “off-label use” of the contrast agent being applied intratympanically.

### Intratympanic Application of the Contrast Agent

Gadoteric acid with a concentration of 279.3 mg/ml is diluted 1:10 with a sodium chloride 0.9% isotonic solution. Around 20 min prior to intratympanic administration, a solution of 10% lidocaine is applied to the tympanic membrane and the auditory canal, providing fully coverage (e.g., by pump spray or directly poured). Lidocaine will be completely removed by suction, subsequently. A volume of 0.4–0.5 ml of the diluted gadoteric acid is then administered through the anterior/superior quadrant of the tympanic membrane into the middle ear. Patients remain lying with their head turned to the contralateral side for 30 min after application and are advised not to speak to reduce an efflux of contrast fluid through the Eustachian tube. The MRI is performed the following day, but within 24 h after application of the contrast agent.

An audiometric control to exclude potential harm on inner ear function through the contrast agent or noise-exposure through MRI is performed routinely within 7 days of examination.

### MRI Technique and Evaluation Process

**Figure 1:**
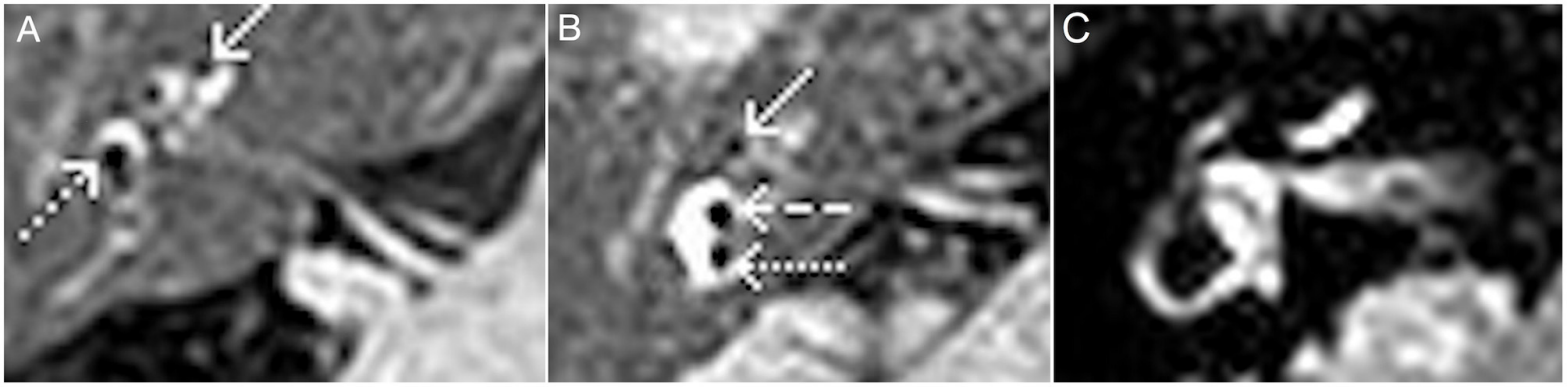
MRI (inversed recovery) of a right inner ear, axial plane. **(A)** Evidence of cochlear (arrow) and vestibular hydrops (dotted arrow). Endolymphatic space is clearly visible by its hypointense (dark) signal, compared to the hyperintense (bright) signal of the contrast-enhanced perilymphatic space. **(B)** Evidence of cochlear (arrow) and vestibular hydrops; saccule (dashed arrow) and utricle (dotted arrow). **(C)** Regular contrast enhanced perilymphatic space of vestibule and cochlea; no enlargement of endolymphatic space.

Radiological evaluation is performed with a 3 Tesla MR machine (Achieva, Philips Medical Systems™). Interpretation of all imaging results have been carried out by the same radiologist—author A.S. The acquired sequences are a coronal fluid attenuated inversion recovery (FLAIR, slice thickness 4 mm), an axial T1 (2.0 mm) and diffusion weighted imaging (DWI, slice thickness 4 mm, b:0, b:1000, ADC maps) over the whole neurocranium for general diagnostic purposes (i.e., to exclude stroke or other cerebral pathologies). An isometric post contrast axial T1 black blood sequence (voxel size 0.8 mm) is performed for general diagnostic purposes (i.e., to exclude vestibular schwannoma) and for the inner ear an isometric axial T2 (1.0 mm voxel size). Our diagnostic “Menière” sequence is an isometric inversion recovery sequence (T1 weighted, voxel size 0.8 mm, TE: 354 ms, TR: 7,600 ms), which shows contrast enhancement of the perilymphatic structures of the vestibular system and the cochlea after intratympanic contrast agent administration. ELH is displayed indirectly as peripherally displaced or missing enhancement of the structures (Figure 1). The radiologist reports on the likeliness of ELH and if presumed positive, the anatomical sites affected. In order to provide comprehensible data, imaging results were reviewed and in case of ELH, the grading system of Baráth et al. (9) was applied to the results (Table 1).

**Table 1:** Patient's individual symptoms and diagnostic results; grading according to Baráth et al.

**Nr**.	**Age**	**Sex**	**ELH in MRI**	**Location & grading of ELH**	**Index ear**	**Vertigo**	**Aural fullness**	**Tinnitus**	**SNHL**	**FQ**	**vHIT**	**Caloric response**	**cVEMPs**
1	76	f	yes	coch I/vest I	R	spinning	none	none	R	all	normal	R <	R <
2	68	m	yes	coch II/vest II	L	spinning	L	L	L	all	L <	symmetric	inconclusive
3	64	m	yes	coch I/vest I	L	none	none	L	L	low	normal	L <	L <
4	61	m	yes	coch II/vest II	L	spinning	L	L	L	all	normal	L <	L <
5	48	f	yes	coch I/vest I	R	swaying	R	R	R	all	normal	R <	symmetric
6	43	f	none	None	L	swaying + spinning	none	L	L	all	normal	L <	not done
7	42	f	none	None	L	spinning	none	L	L	low	normal	L <	not done
8	36	m	yes	coch I/vest I	R	swaying	R	R	R	low	normal	R <	R <
9	34	f	none	None	R	swaying + spinning	R	R	R	low	normal	R <	symmetric
10	73	m	none	None	L	spinning	None	L	B, L < R	low + high	normal	L <	L <
11	66	m	none	None	L	spinning	L	L	L	low	normal	L <	L <
12	57	m	none	None	R	spinning	R	R	R	low	normal	R <	L <
13	44	f	none	None	R	daze feeling	R	R	R	low	normal	R <	not done

*f, female; m, male; ELH, endolymphatic hydrops; MRI, magnetic resonance imaging; coch cochlea, vest vestibule, SNHL, sensorineural hearing loss; FQ, frequencies affected from SNHL, vHIT video head impulse test, cVEMPs cervical vestibular evoked myogenic potentials; R, right; L, left; B, bilateral, < decrease in comparison to contralateral side*.

## Results

Since 2017 we performed endolymphatic hydrops imaging (EHI) in 13 patients (6 female, 7 male), aged between 34 and 76 (mean 54.7 years) at the time of examination. Median duration of symptoms was 12 months. Mean pure-tone average for frequencies 500, 1,000, 2,000 and 4,000 Hz on the index ear was 43.5 dB HL. ELH was detected in six patients (46.2%). In seven patients (53.8%), no sign of ELH was apparent. In one case, analysis could not be performed appropriately due to technical problems. In this specific case the “black-blood sequence” was analyzed but showed no sign of ELH. Intratympanic application of contrast agent has been well-tolerated by all patients. Except slight, self-limiting vertigo during or shortly after its application in some patients, no side effects have been reported. Audiometric follow-up, around 7 days after intervention, showed no worsening of hearing thresholds. In all confirmed cases, ELH was located in both, the cochlea and the vestibule.

Referring to the proposed classification from Gürkov et al. (5, 6) for HED, we assigned the six patients with confirmed ELH to a group based on their individual anamnesis and symptoms. Consequently, five patients were classified as certain primary endolymphatic hydrops (PHED) of the cochleo-vestibular type and one patient as the cochlear type. In the group without confirmed ELH, six patients showed symptoms appropriate to the cochleo-vestibular type and one patient to the cochlear type. Table 1 shows a summary of the patients' individual symptoms and clinical data.

## Discussion

We consider the right selection of patients for endolymphatic hydrops imaging (EHI) as crucial for meaningful results. Until now, we performed EHI in selected patients with unclear, recurring vestibular and/or cochlear symptoms, where allocation of symptoms to a specific disease was not possible on a sole clinical basis. In accordance with recent literature (6) we try to avoid nomenclature like “atypical” MD, by focusing on the potential pathophysiological background with the help of EHI.

We did not define a specific period of symptom duration before undertaking EHI, yet. To date, we are performing EHI only for initial diagnosis and do not use it for follow-up examinations. Moreover, in patients with typical symptoms of MD, who already had a cerebellopontine angle MRI, we do not repeat specific EHI. If MRI results are not present thus far (and in general every patient with “idiopathic” vestibular or cochlear symptoms should get an MRI at least once), EHI can be added with the patient's approval.

The established methods for contrast agent administration are the intravenous or intratympanic application. We prefer the intratympanic application due to its better uptake into the perilymphatic space and therefore, improved radiological assessment (10). Moreover, potential gastro-intestinal adverse effects or failure of renal function reported after systemic, intravenous application can be precluded through local, intratympanic administration. Disadvantages of this method compared to the intravenous application are the additional time and effort needed, the inconvenience for the patient and that this procedure is classified as an off-label use. Moreover, if both ears are to be examined, bilateral intratympanic application of the contrast agent is necessary. Even though this procedure can be useful to detect an asymptomatic ELH on the contralateral side or to evaluate a patient with symptoms in both ears, we follow the approach emphasized by a recent study of Gürkov et al. (11) and strictly perform EHI unilaterally, namely on the more affected side, the so called “index ear.” Despite the off-label use of intratympanic application of gadoteric acid, we have not found any reports in literature, nor did we experience any severe side effects in our patients thus far (12, 13).

A multicenter evaluation of Pyykkö et al. demonstrated ELH in 90% of patients with typical symptoms of MD and even 75% of patients with unilateral symptoms showed bilateral ELH in EHI. In monosymptomatic patients ELH was detected in 55–90% (10). In our group, about half of the patients did not show ELH in the MRI. The difference in numbers might originate from the time point when EHI was performed. Our patients were asymptomatic at the time the MRI was performed and the moment to detect ELH may have passed. In a report by Shi et al. (14), EHI was consistently performed 1 week after the patient suffered from acute symptoms like a vertigo attack or hearing loss. The authors report of a detection rate for ELH of 100% in the affected ear in 198 patients. Therefore, it may be ideal to perform EHI directly after onset of symptoms, but in the clinical routine fast availability of MRI for “elective indication” is not always possible. This may be a potential explanation for the reduced number of detected ELH in our patient group.

One of our patients with suspected MD and a known allergy to gadoteric acid did not receive EHI in consideration of the risk-benefit-ratio and concerning the fact that the procedure is classified as an off-label-use, although it seems unlikely that the local application of a small amount of contrast agent into the middle ear would cause a systemic allergic reaction compared to an intravenous application.

The quality of the evaluation of the images highly depends on the radiologist's skills and experience. A possible source of error which influences the quality can be an insufficient application of the contrast agent, like insufficient filling of the middle ear. Other mitigating factors are swallowing, speaking, or moving the head, which leads to a faster elimination of contrast agent from the middle ear cavity. A difference in the patient's individual permeability of the round window (e.g., after infections, surgery etc.) may also account for diverging results. Currently, a duration of 24 h after intratympanic application of gadoteric acid seems to be the optimal scan time for EHI (15). Previous studies have shown a significant correlation between low-frequency hearing thresholds and the grade of ELH, otherwise no correlation have been found between ELH and the severity of vestibular symptoms (e.g., frequency of vertigo attacks) (9, 14). In our group, two of six patients with verified ELH had low-frequency hearing loss, while in the remaining four all frequencies were decreased. However, all patients without confirmed ELH showed at least low-frequency hearing loss on the index ear. In case of negative EHI, a control MRI could be considered subsequently, e.g., if progression of symptoms occurs. To date, we do not have a clear recommendation regarding this situation.

### Personal View on the Topic

In our opinion EHI is a very beneficial procedure, which improves the diagnostic workup of various inner ear dysfunctions in patients with suspected ELH. Proof of ELH by EHI helps to remove uncertainties and visualize the cause of the disease, therefore, making it more comprehensible for patients and healthcare providers alike. Subsequently, patients are better equipped to understand and accept their disease. The psychological strain of patients, who suffer from an unknown or suspected disease, can be reduced by making a clear diagnosis, which can prevent repeated consultations and examinations (“doctor shopping”). With a diagnosis and the visualization of its cause, it is easier to convey therapeutic measures to the patient, especially in case of destructive methods (e.g., gentamicin). For the previous 3 years, we have performed EHI only with intratympanic application of gadoteric acid and considering the lack of side effects, as well as the improved radiological assessment, we do not see a clear benefit for implementing the intravenous method.

Our current number of cases is small, but we are highly motivated to expand the usage of this diagnostic procedure. Due to its recent and ongoing development we think that in the future the value of this imaging technique will increase, not only for patients with MD like symptoms, but also for other clinical entities, which are associated with the endolymphatic system (e.g., enlarged vestibular aqueduct). Finally, we want to emphasize our clear belief in the scientific value of this method, potentially contributing to the clarification of unanswered questions within the field.

## Conclusion

Endolymphatic hydrops imaging is a valuable method for the diagnostic workup in patients with recurring vestibular and auditory symptoms as described in MD, but inconclusive clinical examination results. In these cases diagnosis of MD or HED, respectively, can be based on the additional presence of ELH in EHI. As an early diagnosis of MD or HED can lead to more specific treatment and less costs for further diagnostics in the long run, we think that EHI should be performed more frequently during primary assessment than has previously been the norm. However, the indication to perform EHI and its final evaluation of results rely on initially thorough vestibular diagnostics. Open questions, like an optimum point in time for EHI, the potential value of repeatedly performed investigations for follow-up examinations or treatment evaluation, as well as the interpretation of missing hydrops in cases of distinct clinical findings need to be addressed in the future.

## Data Availability Statement

The raw data supporting the conclusions of this article will be made available by the authors, without undue reservation.

## Ethics Statement

Ethical review and approval was not required for the study on human participants in accordance with the local legislation and institutional requirements. Written informed consent for participation was not required for this study in accordance with the national legislation and the institutional requirements.

## Author Contributions

LW and SR established idea and content of the article. AO‘S and LW collected and analyzed data. AS provided technical information. All authors contributed equally to this work and discussed results and contributed to the manuscript at all stages.

## Conflict of Interest

The authors declare that the research was conducted in the absence of any commercial or financial relationships that could be construed as a potential conflict of interest.

## Publisher's Note

All claims expressed in this article are solely those of the authors and do not necessarily represent those of their affiliated organizations, or those of the publisher, the editors and the reviewers. Any product that may be evaluated in this article, or claim that may be made by its manufacturer, is not guaranteed or endorsed by the publisher.
